# A retrospective analysis of risk-reducing salpingo-oophorectomy performed in women diagnosed with hereditary breast and ovarian cancer at our institution

**DOI:** 10.1186/s13053-026-00335-0

**Published:** 2026-03-21

**Authors:** Yusaku Shimizu, Miho Kitai, Masashi Akada, Michihide Maeda, Eri Yamabe, Reisa Kakubari, Tsuyoshi Hisa, Shoji Kamiura

**Affiliations:** 1 Department of Gynecology, Osaka International Cancer Institute, Osaka, Japan; 2 Department of Obstetrics and Gynecology, Ashiya Municipal Hospital, Hyogo, Japan

**Keywords:** Hereditary breast and ovarian cancer, Risk-reducing salpingo-oophorectomy, Serous tubal intraepithelial carcinoma, High-grade serous carcinoma

## Abstract

**Background:**

Hereditary breast and ovarian cancer (HBOC) confers a markedly increased lifetime risk of breast and ovarian cancers. As no effective surveillance method for early detection of ovarian cancer has been established, risk-reducing salpingo-oophorectomy (RRSO) is recommended for patients with HBOC to prevent disease onset. We evaluated the clinical characteristics of patients with HBOC and the surgical outcomes of RRSO performed at our institution.

**Methods:**

We retrospectively reviewed women diagnosed with HBOC at our institution between 2018 and 2024. For analyses of surgical outcomes, male patients, patients with prior bilateral adnexectomy, and patients diagnosed with HBOC who did not undergo RRSO were excluded. Clinical data including patient characteristics, surgical procedures, genetic testing, and pathological findings were assessed.

**Results:**

A total of 283 women were diagnosed with HBOC, and 43 (15.1%) underwent testing based on the results of affected relatives. After excluding 39 patients with prior bilateral adnexectomy, and 105 who did not undergo RRSO, 139 patients were included in the surgical analysis. The uptake rate of RRSO among female patients with HBOC was 57%. The median age at surgery was 50 years (range, 35–75). Pathogenic *BRCA1* variants were identified in 48 patients (34.5%), *BRCA2* variants in 90 (64.7%), and both *BRCA1* and *BRCA2* variants in 1 (0.7%). A history of malignancy was observed in 121 patients (breast cancer, *n* = 121; pancreatic cancer, *n* = 1; ovarian cancer, *n* = 1; others, *n* = 3). Laparoscopic RRSO was performed in 138 cases, with one additional hysterectomy performed laparoscopically and one via laparotomy. No perioperative complications were observed. All 6 cases before April 2020 were performed outside insurance coverage; after insurance coverage was initiated in April 2020, 16 were non-covered and 117 were covered by insurance. Pathological examination revealed occult high-grade serous carcinoma in 4 patients (2.9%) and serous tubal intraepithelial carcinoma in 5 patients (3.6%). To date, no cases of primary peritoneal carcinoma following RRSO have been identified.

**Conclusions:**

RRSO was performed safely at our institution, with the detection rate of intraepithelial and invasive carcinoma comparable to previous reports. Although no cases of primary peritoneal carcinoma have been observed postoperatively to date, the residual risk remains, indicating the need for continued long-term surveillance.

## Background

Hereditary breast and ovarian cancer (HBOC) is a hereditary tumor syndrome primarily caused by germline pathogenic variants (PV) in *BRCA1* or *BRCA2* that follows an autosomal dominant inheritance pattern [1]. Patients with HBOC have an increased risk of developing breast and ovarian cancers, accounting for approximately 5% of all breast cancers and 15% of all ovarian cancers [1–3]. Recent large-scale case-control studies have also revealed associations between HBOC and several other malignancies, including prostate, pancreatic, biliary tract, esophageal, and gastric cancers [4]. The reported cumulative cancer risks by the age of 85 years in *BRCA* PV carriers are as follows: among *BRCA1* PV carriers, the breast cancer risk is 72.5% (95% CI, 20.4%–90.5%) and ovarian cancer risk is 65.6% (95% CI, 12.8%–86.4%); among *BRCA2* PV carriers, the breast cancer risk is 58.3% (95% CI, 38.3%–71.9%) and ovarian cancer risk is 14.8% (95% CI, 4.6%–23.9%) [4].

While the risk of ovarian cancer is high in *BRCA1/2* PV carriers, unlike breast cancer, no effective screening method for early detection of ovarian cancer has been established, and most cases are diagnosed at an advanced stage, posing a major clinical challenge. Therefore, risk-reducing salpingo-oophorectomy (RRSO) at an appropriate age is recommended for *BRCA1/2* PV carriers as a preventive measure against ovarian cancer. Compared with non-surgical management, RRSO has been reported to significantly reduce the risk of ovarian cancer and improve the overall survival of patients with HBOC (HR: 0.32; 95% CI: 0.19–0.54; *p* < 0.001) [5]. The National Comprehensive Cancer Network (NCCN) guidelines recommend RRSO for *BRCA1* PV carriers between the ages of 35 and 40 and for *BRCA2* PV carriers between the ages of 40 and 45, after completion of childbearing [6]. This recommendation is based on the observation that ovarian cancer develops 8–10 years later in *BRCA2* PV carriers compared with *BRCA1* PV carriers. In the absence of early-onset cases within the family, delaying the procedure may also be considered. The standard RRSO procedure involves minimally invasive surgery, allowing thorough inspection of the abdominal cavity and complete removal of the ovaries and fallopian tubes [1, 6]. At our institution, RRSO was first introduced as self-financed treatment in 2018, and the number of procedures has increased since its coverage under the Japanese national health insurance system in 2020. However, comprehensive evaluations addressing the surgical safety, detection of occult lesions, appropriateness and timing of RRSO, and the coordination with genetic services and other specialties such as breast surgery remain limited.

In this study, we evaluated the surgical outcomes and clinical significance of RRSO in women with HBOC at our institution through a retrospective review incorporating preoperative genetic testing and postoperative pathological data.

## Material and methods

This was a retrospective study of women diagnosed with HBOC at our institution, the Osaka International Cancer Institute (Osaka, Japan), between January 2018 and December 2024. In this study, HBOC was defined as the identification of a pathogenic or likely pathogenic germline *BRCA1/2* variant on genetic testing performed after genetic counseling. Patients in whom only a variant of uncertain significance (VUS) was detected were not classified as having HBOC and were excluded from the analysis. Our institution is a high-volume comprehensive cancer center with an active gynecologic oncology program, in-house genetic counseling and testing, close collaboration with breast surgery, and specialized gynecologic pathology support. For surgical analyses, women with HBOC and a history of prior bilateral adnexectomy were excluded. Eligible patients were categorized according to whether they underwent RRSO, and baseline clinical characteristics and clinical outcomes were compared across the relevant groups.

Clinical data and family histories of malignancy were extracted from medical records. Genetic counseling was provided to *BRCA* PV carriers by a certified genetic counselor or a gynecologic oncologist. For women desiring fertility preservation or patients with treatment-refractory advanced cancers, RRSO was not recommended after consultation with the attending physician. In some cases, hysterectomy for benign uterine disease was performed concurrently with RRSO. Prior to RRSO, all patients underwent transvaginal ultrasonography and measurement of serum cancer antigen 125 (CA125).

RRSO was performed primarily via minimally invasive surgery. The procedure involved thorough inspection of the abdominal cavity from the upper abdomen to the pelvis, peritoneal washing cytology using 50 mL of saline, resection of the proximal 2 cm of the infundibulopelvic ligament, and complete removal of the fallopian tubes from the uterine cornu, ovaries, and overlying peritoneum. The specimens were retrieved using a collection bag. Pathological evaluation followed the Sectioning and Extensively Examining the Fimbriated End (SEE-FIM) protocol, and immunohistochemical staining for p53 was performed when atypical cells were identified. For patients with a history of breast cancer, risk-reducing mastectomy (RRM) was performed simultaneously with RRSO in collaboration with the breast surgery team when requested. Postoperative surveillance for primary peritoneal carcinoma was conducted every 6–12 months.

The uptake rate of RRSO was calculated as the number of patients who underwent RRSO divided by the number of eligible *BRCA* PV carriers in the study cohort (excluding those with prior bilateral adnexectomy).

Continuous variables are expressed as medians and were compared using the Mann–Whitney U test, while categorical variables were compared using the chi-square test. This study was approved by the Ethics Committee of the Osaka International Cancer Institute (approval number: 24187).

## Results

A total of 283 female patients diagnosed with HBOC were included in the analysis, comprising 93 patients (32.9%) with a *BRCA1* PV and 190 patients (67.1%) with a *BRCA2* PV. The median age at HBOC diagnosis was 49 years in both group (BRCA1: range, 21–78; BRCA2: range, 25–75), with no statistically significant difference between the groups (*p* = 0.551). In both groups, the largest number of patients underwent genetic testing between the ages of 45 and 54 (Table 1).

**Table 1:** Characteristics of the patients

	All *n* = 283	BRCA1 (dual BRCA1/2 mutations) *n* = 93	BRCA2 *n* = 190	p value
Age at HBOC diagnosis, years: median (range)	49 (21–78)	49 (21–78)	49 (25–75)	0.551
<45	89	29	60	
≥45	194	64	130	
History of breast cancer	204	60	144	0.035
Family history of malignancy	255	84	171	1.000
Breast cancer	175	52	123	0.195
Ovarian cancer	77	34	43	0.032
Pancreatic cancer	50	15	35	0.615

HBOC: hereditary breast and ovarian cancer; RRSO: risk-reducing salpingo-oophorectomy; STIC: serous tubal intraepithelial carcinoma; HGSC: high-grade serous carcinoma

Among them, 43 women (15.1%) had not developed malignancy and underwent genetic testing because of results of affected relatives.

A history of breast cancer was observed in 204/283 patients (72.1%) and was more frequent in the BRCA2 group than in the BRCA1 group (BRCA2: 144/190 [75.8%] vs. BRCA1: 60/93 [64.5%]; *p* = 0.035). A family history of malignancy was present in 255/283 (90.1%). Regarding specific family histories, breast cancer was reported in 175/283 (61.8%), with no between-group difference (*p* = 0.195). In contrast, a family history of ovarian cancer was more common in the BRCA1 group (BRCA1: 34/93 [36.6%] vs. BRCA2: 43/190 [22.6%]; *p* = 0.032). A family history of pancreatic cancer was present in 50/283 (17.7%), with no significant difference (*p* = 0.615).

**Fig. 1:**
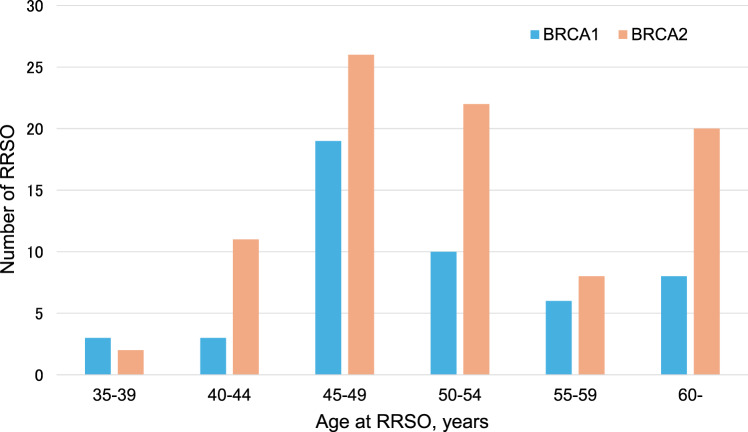
Selection of the study participants. HBOC: hereditary breast and ovarian cancer; RRSO: risk-reducing salpingo-oophorectomy

For the surgical analysis, 39 women with prior bilateral adnexectomy were excluded, leaving 244 eligible women. Among these, 105 patients did not undergo RRSO and 139 patients underwent RRSO and were included for the final surgical analysis (Fig. 1).

The overall uptake rate of RRSO among the 244 female patients with HBOC was 57.0% (139/244). Among the 139 women in the RRSO group, 48 (34.5%) carried a *BRCA1* PV, 90 (64.7%) carried a *BRCA2* PV, and 1 (0.7%) carried both. Uptake was significantly higher among *BRCA1 PV* carriers (68.0%, 49/72; *p* = 0.032) compared with *BRCA2* PV carriers (52.3%, 90/172; *p* = 0.024) (Table 2). The median age at HBOC diagnosis was higher in the RRSO group than in the non-RRSO group (49 years, range 31–65 vs. 42 years, range 21–78; *p* < 0.001). A history of breast cancer was also more frequent in the RRSO group (87.1%, 121/139) compared with the non-RRSO group (69.5%, 73/105; *p* < 0.001). Other prior malignancies included pancreatic (*n* = 1), ovarian (*n* = 1), colorectal (*n* = 2), and lung cancer (*n* = 1). One women with a history of ovarian cancer underwent unilateral adnexectomy and was diagnosed with ovarian cancer, but declined additional surgery. She subsequently developed breast cancer and diagnosed with HBOC; the patient later underwent RRSO. In addition, no women in the non-RRSO group developed ovarian cancer during the screening period (median observation period, 1380 days [range, 493–2793 days]).

**Table 2:** Comparison between the patients undergoing RRSO and surveillance

	RRSO *n* = 139	Non-RRSO *n* = 105	p value
Germline BRCA mutation			
BRCA1	48	23	0.032
BRCA2	90	82	0.024
BRCA1/2	1	0	
Age at HBOC diagnosis, years: median (range)	49 (31–65)	42 (21–78)	<0.001
<35	3	22	
35–44	25	37	
45–54	69	23	
55–64	28	15	
>65	14	8	
History of breast cancer	121	73	<0.001
Family history of malignancy	131	88	0.008
Breast cancer	102	56	0.001
Ovarian cancer	41	24	0.245
Pancreatic cancer	23	19	0.751

RRSO: risk-reducing salpingo-oophorectomy; HBOC: hereditary breast and ovarian cancer

In RRSO group, 94.2% (131/139) had a family history of malignancy, including breast cancer in 102 patients, ovarian cancer in 41, and pancreatic cancer in 23. A significant difference was observed particularly for family history of breast cancer (*p* = 0.001).

**Fig. 2:**
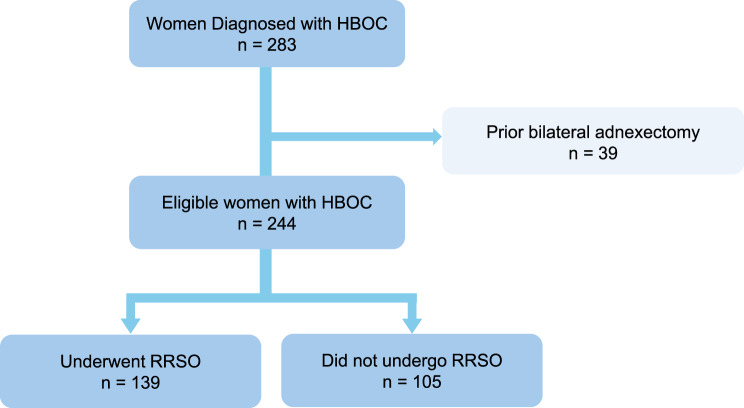
Distribution of RRSO in patients with BRCA pathogenic variants by age

The median age at RRSO was 49 years, with the majority of procedures performed between ages 45 and 49 (Fig. 2). RRSO was not performed in any women before the age of 35.

All six procedures conducted during the pre-insurance period (2018–2020) were self-funded. After insurance coverage was introduced in April 2020, the 117 procedures were covered by the Japanese national health insurance system, whereas the remaining 16 procedures were performed as self-financed procedures in unaffected women who elected to undergo RRSO outside the insurance system.

Of the 139 RRSO procedures, 138 (99.3%) were completed laparoscopically with no conversions to laparotomy. No perioperative complications were observed. Pathological examination revealed serous tubal intraepithelial carcinoma (STIC) in five patients (3.6%) and occult high-grade serous carcinoma (HGSC) in four patients (2.9%). The perioperative and pathological findings stratified by the presence or absence of STIC/HGSC are summarized in Table 3. There were no significant differences in operative time or blood loss between patients in the benign and STIC/HGSC groups. The interval between genetic diagnosis and RRSO was 6 months (range, 1–53) in patients without atypical lesions and 7 months (range, 1–24) in those with STIC/HGSC, with no significant difference (*p* = 0.898). Concurrent contralateral RRM in collaboration with breast surgeons was performed in 51 women (36.7%) upon request, and no perioperative complications were observed in these combined procedures.

**Table 3:** Comparison of perioperative outcomes between patients diagnosed with benign lesions and those diagnosed with STIC/HGSC

	Benign *n* = 130	STIC/HGSC *n* = 9	p value
Interval from genetic testing to RRSO (mo)	6 (1–53)	7 (1–24)	0.898
Operative time (min)	38–238	52–144	0.135
Estimated blood loss (ml)	5–110	5	0.220
Laparoscopic surgery	129 (99.2%)	9 (100%)	0.792
Concurrent RRM	47 (36.2%)	4 (44.4%)	0.618

STIC: serous tubal intraepithelial carcinoma; HGSC: high-grade serous carcinoma; RRM: risk-reducing mastectomy

Among the five STIC cases, two harbored BRCA1 PV and three harbored BRCA2 PV (Table 4). Among the four HGSC cases, two harbored BRCA1 PV and two harbored BRCA2 PV. All nine women had a history of breast cancer.

**Table 4:** Characteristics of incidental carcinomas detected at RRSO

No.	Pathology	TP 53 status	Age at RRSO	Time from diagnosis to RRSO (mo)	Gene type	History of Breast cancer	Family history of ovarian cancer	CA125 (U/mL)	Ascites at surgery	Overall survival
1	STIC	p53 signature	48	7	BRCA1	(+)	(-)	11	Sus.	44 mo NED
2	STIC	Null type	67	3	BRCA2	(+)	(-)	9	Neg.	44 mo NED
3	STIC	p53 signature	53	24	BRCA2	(+)	(+)	-	Neg.	4 mo NED
4	STIC	p53 signature	47	5	BRCA1	(+)	(-)	7	Neg.	21 mo NED
5	STIC	p53 signature	47	1	BRCA2	(+)	(-)	42	Neg.	10 mo NED
6	HGSC	p53 signature	45	8	BRCA1	(+)	(+)	32	Neg.	Olaparib, NED 36 mo
7	HGSC	p53 signature	54	7	BRCA2	(+)	(-)	10	Neg.	PFS 33 mo
8	HGSC	p53 signature	46	8	BRCA1	(+)	(-)	53	Pos.	Olaparib, NED 23 mo
9	HGSC	Null type	60	6	BRCA2	(+)	(-)	7	Neg.	PFS 11 mo

RRSO: risk-reducing salpingo-oophorectomy; STIC: serous tubal intraepithelial carcinoma; HGSC: high-grade serous carcinoma; Sus.: suspicious; Neg.: negative; Pos.: positive; NED: no evidence of disease; PFS: progression-free survival

During the median follow-up of 21 months, none of the patients diagnosed with STIC developed primary peritoneal carcinoma. All four women diagnosed with HGSC underwent primary debulking surgery in accordance with ovarian cancer treatment guidelines, and their disease remains controlled. Concurrent hysterectomy for benign uterine disease was performed in two cases (one laparoscopic, one open), with no findings of endometrial atypical hyperplasia or carcinoma.

## Discussion

This study evaluated the clinical characteristics of women with HBOC at our institution, as well as the surgical outcomes and clinical significance of RRSO. Since the introduction of public insurance coverage in 2020, the number of RRSO procedures has increased nationwide, and a similar trend has been observed at our center. The RRSO uptake rate of 57% observed in this study was comparable to reports from Western countries [7], suggesting that the introduction of insurance coverage has facilitated the acceptance of RRSO in Japan to an internationally comparable level. Notably, the non-RRSO group (surveillance) included patients with treatment-refractory advanced cancers, some of whom had not been referred to gynecology. In addition, many women in the non-RRSO group were younger than 44 years, and considering age and childbearing plans, a substantial proportion were likely awaiting RRSO and had not yet reached an appropriate time for the procedure. Therefore, if the uptake rate were calculated using only patients who were eligible in terms of age and timing as the denominator, the true uptake of RRSO at our institution might be higher than that estimated in this study.

In our HBOC cohort, *BRCA2* PV carriers accounted for the majority (190/283, 67.1%). In addition, a history of breast cancer was more frequent in the BRCA2 group than in the BRCA1 group (BRCA2: 144/190 [75.8%] vs. BRCA1: 60/93 [64.5%]; *p* = 0.035, Table 1). Collectively, these findings indicate that our institutional cohort was characterized by a predominance of BRCA2 carriers and a higher prevalence of prior breast cancer among BRCA2 carriers. Japanese registry data have shown that *BRCA1*-associated breast cancer is more frequently triple-negative breast cancer (TNBC), whereas BRCA2-associated breast cancer more commonly exhibits a luminal phenotype (BRCA1: TNBC 75.8%, luminal 21.2%; BRCA2: TNBC 18.6%, luminal 64.4%) [8]. Such differences in subtype distribution may influence referral patterns and the clinical pathways leading to genetic testing, thereby affecting the observed BRCA1/BRCA2 composition in single-center cohorts. In addition, a high frequency of the *BRCA2* c0.5576_5579del variant has been reported in a neighboring region of our center, suggesting that regional variation in PV prevalence may have influenced the composition of our cohort [9].

Taken together, the predominance of BRCA2 PV carriers in our center may reflect both case ascertainment driven by breast cancer care (i.e., an institutional case mix that mirrors the distribution of breast cancer subtypes) and regional enrichment of specific BRCA2 PV. Future studies incorporating breast cancer subtypes and detailed variant profiles are warranted to clarify the contribution of these factors.

Women carrying *BRCA1/2* PV are at increased risk of ovarian cancer, with *BRCA1* PV carriers reported to have a two- to three-fold higher risk compared with *BRCA2* PV carriers [10]. A family history of ovarian cancer further increases this risk [11], and in our study, such a history was significantly more common among *BRCA1* PV carriers. This likely reflects the appropriate referral and genetic counseling for ovarian cancer patients at our institution. In contrast, a previous systematic review reported no association between family history of ovarian cancer and the uptake of RRSO [12]. A similar trend was observed at our institution. Prior bereavement as a result of gynecologic or breast cancer among family members has been reported to be associated with patients’ decisions to undergo RRSO [13]. These findings suggest that future investigations should incorporate not only the family history of malignancy but also psychosocial factors such as experiences of cancer-related bereavement.

RRSO before the age of 45 has been shown to reduce ovarian cancer-specific mortality among *BRCA1/2* PV carriers [6]. However, premenopausal oophorectomy results in estrogen deficiency, raising concerns regarding long-term health risks such as cardiovascular disease, cognitive decline, and osteoporosis. A large Canadian cohort study reported an increased all-cause mortality associated with oophorectomy before the age of 50 (age < 45: HR 1.31, 95% CI 1.18–1.45, *p* < 0.001; age 45–49: HR 1.16, 95% CI 1.04–1.30, *p* = 0.007), particularly attributable to non-cancer-related deaths, whereas no such association was observed in women ≥ 50 years [14]. Thus, the timing of RRSO requires careful, individualized consideration. In our study, the median age at RRSO was higher than NCCN guideline recommendations [6]. However, detailed follow-up regarding the management of surgical menopause was not available and remains an important issue for future research. Hormone replacement therapy is commonly used to mitigate the adverse effects of surgical menopause, but it is contraindicated in women with a history of breast cancer. Given that approximately 90% of women who underwent RRSO in our study had already developed breast cancer prior to their HBOC diagnosis, the timing of RRSO should not be uniformly dictated by guideline recommendations but rather tailored to each patient, taking into account breast cancer history and the potential consequences of premature menopause.

In our series, RRSO was performed safely, with no conversions to laparotomy and no major perioperative complications. Because the presence of STIC/HGSC cannot be reliably predicted preoperatively, we compared perioperative outcomes between women with benign pathology and those in whom STIC/HGSC was incidentally detected to determine whether unexpected STIC/HGSC adversely affected surgical performance. As a result, no significant differences were observed in operative time, blood loss, or other perioperative parameters, supporting that standard minimally invasive RRSO can be performed safely regardless of postoperative pathological findings. Nevertheless, because a certain proportion of women are found to have STIC/HGSC at the time of RRSO, the SEE-FIM protocol and appropriate postoperative follow-up are essential.

The prevalence of occult HGSC (2.9%) and STIC (3.6%) in our study was consistent with previous Japanese reports (HGSC, 3.2%–10.0% [15, 16]; STIC, 0.6–7% [17]), highlighting that a certain proportion of invasive carcinoma is detected at the time of RRSO. Notably, one women was diagnosed with HGSC at the age of 45. Previous studies have also reported cases of occult carcinoma detected in women in their early forties, and the risk of ovarian cancer rises sharply around the age of 40 in *BRCA1/2* PV carriers [4]. In one case, peritoneal washing cytology was positive and peritoneal dissemination was identified intraoperatively. Adequate inspection of the entire abdominal cavity, including the upper abdomen, bowel surfaces, omentum, and appendix, as well as peritoneal biopsy when indicated, is essential during RRSO. However, there is currently no consensus regarding management when positive peritoneal cytology or peritoneal dissemination is detected intraoperatively [1]. Preoperative MRI or PET-CT for all patients is neither feasible nor supported by evidence. At our institution, preoperative evaluation was limited to transvaginal ultrasonography and CA125 measurement, and RRSO was performed in the absence of suspicious findings. The aforementioned case demonstrated a mildly elevated CA125 (53 U/mL) but no significant ultrasound findings and RRSO was performed without additional imaging. After the pathological diagnosis of malignancy, further imaging was undertaken, followed by radical surgery and adjuvant chemotherapy, resulting in disease-free survival to date. Although CA125 alone is insufficient for diagnosing peritoneal carcinoma, additional imaging studies should be considered when abnormal values are detected, as this may enable earlier therapeutic intervention.

RRSO is an effective preventive measure for ovarian, fallopian tube, and peritoneal cancers, but a residual risk of primary peritoneal carcinoma remains, estimated at 1%–4.9% after RRSO [18–21]. STIC is a precursor lesion that can exfoliate early from the fimbrial epithelium, implant on the peritoneum or ovary, and progress to invasive carcinoma [22]. For this reason, STIC is classified as stage IA fallopian tube carcinoma in the Japanese clinical practice guidelines [23]. A meta-analysis reported that the risk of peritoneal carcinoma is significantly higher in patients with STIC than in those without STIC (HR 33.9, 95% CI 15.6–73.9). The 5- and 10-year cumulative incidences of peritoneal carcinoma were 10.5% (95% CI 6.2–17.2) and 27.5% (95% CI 15.6–43.9), respectively, in patients with STIC compared with 0.3% (95% CI 0.2–0.6) and 0.9% (95% CI 0.6–1.4) in those without STIC [24]. The median interval from RRSO to peritoneal carcinoma onset was reported as 54.5 months overall and 48 months (range, 18–118) in patients with STIC [25]. While no new cases of peritoneal carcinoma have been observed in our series to date, the relatively short follow-up underscores the need for long-term surveillance.

Concurrent RRSO and RRM has recently attracted attention as a strategy to reduce psychological burden, social constraints, and healthcare costs. Previous studies have shown no increase in postoperative complications with combined surgery [26, 27]. Advantages of this approach include the completion of both procedures under a single anesthesia, shorter hospitalization and recovery, and reduced cost. Since physical, psychological, and social burdens affect decision-making regarding preventive surgery, simultaneous procedures may improve the acceptance of risk-reducing interventions. At our institution, simultaneous surgery was performed in collaboration with anesthesiology and breast surgery upon patient request, with no increase in postoperative complications. These findings support the combined RRSO and RRM as a patient-centered and resource-efficient approach.

In our study, only 2 of 139 patients (1.4%) underwent hysterectomy concurrently with RRSO. No cases of endometrial carcinoma were identified during follow-up in either the RRSO or screening groups. However, an increased risk of endometrial carcinoma in *BRCA* PV carriers has been reported [4, 28], indicating the need for long-term monitoring for this malignancy as well as peritoneal carcinoma. Nevertheless, given the additional cost to patients, concurrent hysterectomy should be considered with caution, with careful selection of candidates and thorough preoperative counseling regarding the risks and benefits.

The strengths of this study include the presentation of single-institution data on RRSO outcomes after the introduction of public insurance coverage. Additionally, the inclusion of concurrent RRM cases reflects real-world multidisciplinary practice in the management of HBOC. However, several limitations should be acknowledged. First, the follow-up period after RRSO was relatively short, which may have led to an underestimation of the incidence of primary peritoneal carcinoma and endometrial carcinoma. Moreover, it was difficult to assess the extent to which the protective effect of RRSO in reducing ovarian cancer risk among HBOC patients might be offset by other health consequences. Furthermore, as this was a single-institution study, potential biases related to institutional characteristics may have influenced the results.

## Conclusions

This study clarified the surgical outcomes of RRSO in women with HBOC after the introduction of public insurance coverage in Japan, demonstrating the safety of RRSO and a detection rate of invasive carcinoma and STIC comparable to previous reports. To date, no women under surveillance group have newly developed ovarian cancer, and no cases of primary peritoneal carcinoma or endometrial carcinoma have been observed after RRSO. Nevertheless, the risk of cancer is not completely eliminated after RRSO, and patients with STIC in particular remain at long-term risk of developing peritoneal carcinoma. Thus, while RRSO is an effective preventive intervention, continued and structured postoperative surveillance is essential. Our findings may contribute to improving the quality of HBOC management in Japan and to optimizing the timing and postoperative management strategies of RRSO. Future multicenter collaborative studies in Japan are warranted to accumulate long-term outcome data.

## Data Availability

No datasets were generated or analysed during the current study.

## Abbreviations

*BRCA1*: Breast cancer 1
*BRCA2*: Breast cancer 2
CA125: Cancer antigen 125
HBOC: Hereditary breast and ovarian cancer
HGSC: High-grade serous carcinoma
NCCN: The National Comprehensive Cancer Network
PV: Pathogenic variants
RRM: Risk-reducing mastectomy
RRSO: Risk-reducing salpingo-oophorectomy
SEE-FIM: Sectioning and Extensively Examining the Fimbriated End
STIC: Serous tubal intraepithelial carcinoma
TNBC: Triple-negative breast cancer
VUS: Variant of uncertain significance
